# DNA methylation changes in cord blood and the developmental origins of health and disease – a systematic review and replication study

**DOI:** 10.1186/s12864-022-08451-6

**Published:** 2022-03-19

**Authors:** Loubna Akhabir, Randa Stringer, Dipika Desai, Piush J Mandhane, Meghan B Azad, Theo J Moraes, Padmaja Subbarao, Stuart E Turvey, Guillaume Paré, Sonia S. Anand, for the NutriGen Alliance, Sonia S. Anand, Stephanie A. Atkinson, Meghan B. Azad, Allan B. Becker, Jeffrey Brook, Judah A. Denburg, Dipika Desai, Russell J. de Souza, Milan Gupta, Michael Kobor, Diana L. Lefebvre, Wendy Lou, Piushkumar J. Mandhane, Sarah McDonald, Andrew Mente, David Meyre, Theo J. Moraes, Katherine Morrison, Guillaume Paré, Malcolm R. Sears, Padmaja Subbarao, Koon K. Teo, Stuart E. Turvey, Julie Wilson, Salim Yusuf, Stephanie Atkinson, Gita Wahi, Michael A. Zulyniak

**Affiliations:** 1 Population Health Research Institute, David Braley Cardiac, Vascular and Stroke Research Institute, 237 Barton Street East, Hamilton, ON Canada; 2 Department of Medicine, McMaster University, 1280 Main Street West, L8S 4K1 Hamilton, ON Canada; 3 Department of Pediatrics, University of Alberta, Edmonton, AB Canada; 4 Department of Pediatrics and Child Health, Children’s Hospital Research Institute of Manitoba, University of Manitoba, Winnipeg, MB Canada; 5 Department of Pediatrics, Hospital for Sick Children, ON Toronto, Canada; 6 Department of Pediatrics, BC Children’s Hospital, University of British Columbia, Vancouver, BC Canada; 7 Thrombosis and Atherosclerosis Research Institute, David Braley Cardiac, Vascular and Stroke Research Institute, 237 Barton Street East, Hamilton, ON Canada; 8 Department of Pathology and Molecular Medicine, Michael G. DeGroote School of Medicine, McMaster University, 1280 Main Street West, Hamilton, ON Canada; 9 Department of Clinical Epidemiology & Biostatistics, McMaster University, 1280 Main Street West, Hamilton, ON Canada; 10 Department of Health Research Methods, Evidence, and Impact, McMaster University, Hamilton, Ontario Canada

**Keywords:** Cord blood; methylation; EWAS; prenatal, *In utero*, CHILD, START, Nutrigen

## Abstract

**Background:**

Environmental exposures *in utero* which modify DNA methylation may have a long-lasting impact on health and disease in offspring. We aimed to identify and replicate previously published genomic loci where DNA methylation changes are attributable to *in utero* exposures in the NutriGen birth cohort studies Alliance.

**Methods:**

We reviewed the literature to identify differentially methylated sites of newborn DNA which are associated with the following five traits of interest maternal diabetes, pre-pregnancy body mass index (BMI), diet during pregnancy, smoking, and gestational age. We then attempted to replicate these published associations in the Canadian Healthy Infant Longitudinal Development (CHILD) and the South Asian birth cohort (START) cord blood epigenome-wide data.

**Results:**

We screened 68 full-text articles and identified a total of 17 cord blood epigenome-wide association studies (EWAS) of the traits of interest. Out of the 290 CpG sites reported, 19 were identified in more than one study; all of them associated with maternal smoking. In CHILD and START EWAS, thousands of sites associated with gestational age were identified and maintained significance after correction for multiple testing. In CHILD, there was differential methylation observed for 8 of the published maternal smoking sites. No other traits tested (i.e., folate levels, gestational diabetes, birthweight) replicated in the CHILD or START cohorts.

**Conclusions:**

Maternal smoking during pregnancy and gestational age are strongly associated with differential methylation in offspring cord blood, as assessed in the EWAS literature and our birth cohorts. There are a limited number of reported methylation sites associated in more than two independent studies related to pregnancy. Additional large studies of diverse populations with fine phenotyping are needed to produce robust epigenome-wide data in order to further elucidate the effect of intrauterine exposures on the infants’ methylome.

**Supplementary Information:**

The online version contains supplementary material available at 10.1186/s12864-022-08451-6.

## Background

The Developmental Origins of Health and Disease (DOHaD) is an area of research that investigates the influence of pregnancy and early life exposures on the growth, development, and health of the offspring. Fetal programming is a process whereby the *in utero* environment may prime a fetus for an expected environment after delivery, which may have a significant impact on lifelong health. It is thought that epigenetic mechanisms, in particular DNA methylation, mediate fetal programming [1, 2] and may modify trajectories of health and disease throughout the offspring’s lifetime [1, 3, 4]. Technological advances have enabled expansive evaluations of epigenetic modifications [1] so that researchers are now able to interrogate the epigenome for signs of fetal programming.

Maternal nutrition and chemical exposures are associated with epigenetic changes which may lead to the development of a variety of non-communicable diseases. It is thought that these epigenetic changes are responsible for what is described as the developmental origins of health and disease whereby early life environmental stresses influence life-long disease risk [5]. For instance, the maternal famine exposure in the Dutch Hunger Winter of 1944 to 1945 and Chinese famine of 1959 to 1961, is linked with an increased incidence of type-2 diabetes in the offspring [6]. This phenomenon of “developmental programming” has also been shown in less severe exposures such as in response to a dysglycemic *in utero* environment and has been linked to altered DNA methylation patterns in placental and infant tissues [7, 8]. For instance, differential methylation and gene expression have been observed at the *IGF2/H19* locus, an imprinted region involved in metabolic programming, in the blood samples of offspring exposed to intrauterine hyperglycemia in human and animal studies [9, 10]. Maternal obesity, excess pregnancy weight gain, and gestational diabetes mellitus (GDM) are risk factors for negative health outcomes in the offspring. There is a mounting body of evidence that several other exposures including maternal smoking and nutrition also lead to adverse child health outcomes, potentially through modification of their DNA methylation profile [11–13].

We sought to identify and replicate previously published genomic loci where DNA methylation changes are attributable to *in utero* exposures, including maternal GDM, maternal pre-pregnancy body mass index (BMI), diet during pregnancy, maternal smoking, and to perinatal traits such as gestational age and birthweight.

First, we searched the literature to identify robustly associated methylation sites for these exposures/traits of interest, and second, we attempted to replicate these robust published associations in two independent prospective birth cohort studies of South Asians and white Caucasians in which epigenome-wide data are available.

## Methods

### Literature review

**Fig. 1:**
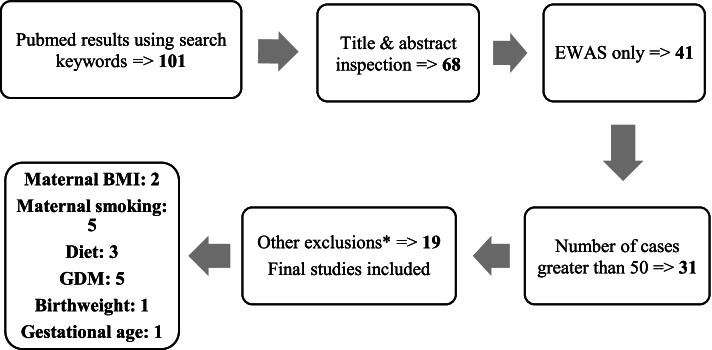
Consort diagram detailing the process of the systematic review

We conducted a generalized search in the PubMed database to identify CpG probes associated with our 5 exposures of interest and perinatal traits: prenatal nutrition (especially dietary patterns), maternal smoking, maternal or infant dysglycemia (especially GDM), gestational age, and maternal pre-pregnancy BMI. The following search was used: “cord blood” AND “DNA methylation” AND “pregnancy” AND (“diet” OR “nutrition” OR “dietary pattern” OR “diabetes” OR “dysglycemia” OR “glucose” OR “insulin” OR “smoking” OR “gestational age” OR “weight” OR “BMI”). The literature search was conducted in January 2020. Figure 1 delineates the steps and articles inclusion.

Inclusion criteria were: (i) studies in cord blood, (ii) studies reporting epigenome-wide associations and (iii) number of cases greater than 50. Targeted studies, those that reported statistically non-significant results, or EWAS in peripheral blood or placental tissue were excluded.

All abstracts were then screened for our inclusion and exclusion criteria, with some studies moving on for full text evaluation and possible inclusion in the review. Our search returned a total of 101 results which were screened at the abstract level. Of these, 68 full-text articles were accessed for closer inspection, and a final of 19 studies were identified for inclusion in our review.

### Study population, EWAS, and Replication Study

The NutriGen study, is an alliance of four birth cohorts, and has been described previously [14]. Briefly, the NutriGen study aims to investigate nutrition and environmental determinants of maternal and childhood health, with a focus on genetic, epigenetic, and microbiome contributions. Cord blood samples were collected at birth for genetic and cardiometabolic analysis. The present report deals with data from two of the NutriGen cohorts in whom EWAS was conducted: the South Asian birth cohort (START) [15] and the Canadian Healthy Infant Longitudinal Development (CHILD) [16] (Additional File 1). Newborn anthropometric information was collected at the time of birth and cord blood collected where possible.

All pregnant women completed a food frequency questionnaire (FFQ) to assess prenatal diet and were assessed for anthropometric and other health measures (Additional File 1). Principal component (PC) analysis of FFQ data was used to derive individual scores representing dietary patterns. Individual nutrient information regarding fatty acid intake was used, including polyunsaturated fatty acids (PUFAs), saturated fats, and the ratio of PUFAs to saturated fats (P:S).

Two diet quality scores, the modified Alternative Healthy Eating Index (mAHEI) and the DOHaD score developed by the NutriGen group, were calculated and used in analysis. Dietary variables were adjusted for total energy consumption when applicable.

These variables have been harmonized across cohorts for comparisons. A harmonized GDM variable was available in both START and CHILD. Maternal smoking and pre-pregnancy BMI were only available in CHILD; whereas in START there were no smokers during pregnancy, and BMI was measured in the second trimester.

### Epigenome Wide Association Study (EWAS)

DNA was extracted from 512 START and 511 CHILD cord blood samples and subjected to bisulfite conversion. The samples were hybridized to the Illumina HumanMethylation450K beadchip array. Raw data generated by the Illumina iScan software were processed using the minfi R package [17]. Quality control and pre-processing were performed in each cohort separately. Samples were excluded if they showed > 0.01 failed probes or had a discrepancy between genetic sex and reported sex. Probes were excluded based on > 0.05 missingness, and if they contained a SNP or showed cross-reactivity [18]. Clean data were obtained for 393,400 probes in 491 START samples and for 393,449 probes in 511 CHILD samples. ReFaCTor reference-free algorithm was used to obtain principal components of cell composition [19, 20]. For CHILD and START cohorts separately, cord blood DNA methylation was assessed for association using multivariate linear regression with the following exposures of interest / prenatal traits: (1) diet, including dietary patterns, macronutrient intake, diet quality scores, (2) gestational age, (3) GDM, and (4) smoking during pregnancy (CHILD only), and pre-pregnancy BMI (CHILD only). The following variables were included as covariates in the association models: maternal age, infant sex, gestational age, study centre, processing time (CHILD only), smoking (CHILD only), and cellular composition. Statistical analyses were conducted in R version 3.2.0. The Bonferroni correction was used to adjust for multiple testing, with a significance threshold of *p* < 1.27 × 10^− 7^. EWAS analysis in CHILD was restricted to the European subset (*N* = 295), due to low sample size from other ethnic groups. Gene set enrichment analysis for gestational age significant results was performed using the R package ‘enrichr’ [21].

### Replication study

Where data were available, we used the START and CHILD DNA methylation to conduct a targeted replication analysis based on the findings of the literature review. Analyses were repeated similarly as in the EWAS using linear regression models, for only those sites identified in the literature as significantly associated with a given outcome.

Multivariate linear regression models were used to test for association between DNA methylation β values at each site and the exposures/traits variables. The association models were adjusted, as appropriate, for factors suspected to influence DNA methylation: maternal age, infant sex, gestational age, study centre, processing time (CHILD only), smoking (CHILD only), and cell composition. The Bonferroni correction was used to adjust for multiple testing, using the number of probes tested for each replication analysis. We adjusted for appropriate covariates for each replication analysis. Specifically, for the CHILD maternal smoking replication analysis, we adjusted for maternal age, sex, gestational age, study centre, cellular composition. For the folate level replication analysis in START, we adjusted for maternal age, sex, gestational age, total homocysteine levels, vitamin B12 levels, RBC folate levels, study centre and cellular composition.

For the birthweight replication in CHILD and START, we adjusted for maternal age, sex, smoking, gestational age, study centre, and cellular composition.

Estimates for diet, birthweight, GDM and gestational age from CHILD and START models were meta-analyzed using the ‘meta’ R package inverse variance method, to obtain fixed and random effect estimates as well as a heterogeneity statistic.

## Results

### Literature review

Using the keywords for traits of interest, we identified 68 studies for which we read the full text publications, 41 of them were EWAS. After excluding studies with less than 50 cases, 31 remained. Nineteen of these studies reported significant methylation results and were included in our analysis.

These studies reported 259 significantly associated DNA methylation probes for the following traits: (1) birthweight (12 probes) and gestational age (22 probes), (2) childhood adiposity (6 probes), (3) GDM (96 probes) and maternal diabetes (16 probes), (4) maternal obesity (3 probes), (5) maternal eating disorder (2 probes), (6) maternal polyunsaturated fatty acids (PUFA) (6 probes) and trans fatty acids (FA) (5 probes) at preconception, (7) maternal plasma folate levels (48 probes) and (8) maternal smoking during pregnancy (60 probes).

The 259 identified sites were significantly associated with their trait after correction for multiple testing. Bonferroni correction was used for correction in 9/18 publications, while 9/18 used FDR. One article reported 18 methylation sites associated with GDM without any multiple testing correction. We added the non-corrected GDM sites to our list of interest for a total number of 277 sites to include in our analysis.

Among the 277 sites, only 19 replicated in at least one other study. All of these were maternal smoking-associated probes. A list of the probes is provided in the Additional Table 1. Additional Table 2 details all studies identified, and the probes extracted.

We also noted that the birthweight publication [22] reported robust findings from a large meta-analysis of EWAS; and we included 12 sites where the association persisted in childhood peripheral blood in our sites to test for replication.

### EWAS

CHILD and START study participants’ characteristics are shown in Table 1.

**Table 1:** Characteristics of CHILD and START EWAS participants

Characteristics	START (South Asians)	CHILD (European subset)
Sample size	491	295
Maternal age (years, mean ± SD)	30.9 ± 3.9	32.7 ± 4.4
Infant sex (% Female)	51.9	46.1
Gestational age (weeks, mean ± SD)	39.2 ± 1.3	39.5 ± 1.3
Birth weight (grams, mean ± SD)	3,265 ± 454.5	3,498.6 ± 485.9
Smoking (N /%)	0 / 0	23 / 7.8

We observed no epigenome-wide significant differential DNA methylation for diet, maternal smoking or maternal GDM, after correction for multiple testing in separate cohort analyses or in a meta-analysis of the cohorts. Gestational age was highly associated with thousands of sites in both cohorts independently. The meta-analysis yielded 5,732 significant associations after Bonferroni correction for multiple testing. Gene set enrichment analyses of the significant results did not yield any statistically significant enriched Gene Ontology (GO) pathway (Additional Table 3). Table 2 indicates the top 10 sites identified in the meta-analysis. All 5,732 significant results are listed in Additional Table 4. A volcano plot of the results is provided as Additional Fig. 1.

**Table 2:** Top 10 sites most significantly associated with gestational age in the meta-analysis of START and CHILD

Site	Chr	Position	Gene	Estimate (CI)	Nominal *P* value	Adjusted *P* value
***cg11932158***	3	155,422,129	*PLCH1*	-0.019 (-0.022, -0.016)	4.8 × 10^− 47^	1.9 × 10^− 41^
***cg18623216***	3	155,421,970	*PLCH1*	-0.021 (-0.024, -0.018)	3.7 × 10^− 46^	1.5 × 10^− 40^
***cg16103712***	8	99,023,869	*MATN2*	-0.023 (-0.026, -0.020)	1.2 × 10^− 45^	4.8 × 10^− 40^
***cg17133774***	1	6,198,667	*CHD5*	-0.018 (-0.020, -0.015)	4.1 × 10^− 45^	1.6 × 10^− 39^
***cg12713583***	19	940,724	*ARID3A*	-0.022 (-0.025, -0.019)	6.3 × 10^− 45^	2.5 × 10^− 39^
**cg04347477**	12	125,002,007	*NCOR2*	-0.019 (-0.022, -0.016)	5.3 × 10^− 41^	2.1 × 10^− 35^
*cg08817867*	17	19,656,554	*-*	-0.018 (-0.020, -0.015)	5.4 × 10^− 39^	2.1 × 10^− 33^
*cg02001279*	19	940,967	*ARID3A*	-0.013 (-0.015, -0.011)	7.7 × 10^− 39^	3.0 × 10^− 33^
**cg08412913**	16	85,429,522	*-*	-0.011 (-0.013, -0.010)	1.3 × 10^− 35^	4.9 × 10^− 30^
cg06870470	19	11,315,767	*DOCK6*	-0.019 (-0.022, -0.016)	8.2 × 10^− 35^	3.2 × 10^− 29^

*Chr *chromosome, *CI *confidence interval; Adjusted p value: Bonferroni-corrected p value; bolded (or italic) CpG sites are also top 10 significant associations in the separate START (or CHILD) analysis; bolded and italic sites were in the top 10 significant associations in both cohort specific analyses. Analyses were adjusted for maternal age, sex, study centre and cellular composition. Estimates are expressed as the change in methylation beta value per one unit change in gestational age in weeks

### Targeted replication

Using the probes identified using the literature review, we conducted replication analyses in START and CHILD for plasma folate levels (48 sites) and maternal smoking (25 sites), respectively. Both single study analyses and meta-analyses were conducted for birthweight (12 sites) and gestational age (279 sites).

Gestational age in CHILD and START meta-analysis was significantly associated with 37% of the sites identified in the literature, all with consistent direction of effects (Additional Table 5).

Eight of the maternal smoking probes showed statistically significant association with the exposure in CHILD, after correction with multiple testing (Table 3). START mothers were all non-smokers.

**Table 3:** Replicated maternal smoking-associated sites in CHILD

Site	Chr	Position	Gene	Estimate (se)	Nominal *P* value	Adjusted *P* value	Literature Estimate (se)	Author (PMID)
cg05549655	15	75,019,143	*CYP1A1*	0.027 (0.006)	3.8 × 10^− 6^	9.4 × 10^− 5^	0.019 (0.001)	Joubert et al. 27,040,690
cg11924019	15	75,019,283	*CYP1A1*	0.043 (0.010)	2.2 × 10^− 5^	5.6 × 10^− 4^	0.025 (0.002)	Joubert et al. 27,040,690
cg22549041	15	75,019,251	*CYP1A1*	0.047 (0.011)	3.5 × 10^− 5^	8.9 × 10^− 4^	0.066 (0.004)	Joubert et al. 27,040,690
cg23067299	5	323,907	*AHRR*	0.036 (0.008)	4.7 × 10^− 5^	0.001	0.030 (0.002)	Joubert et al. 27,040,690
cg22132788	7	45,002,486	*MYO1G*	0.051 (0.013)	7.9 × 10^− 5^	0.002	0.023 (0.001)	Joubert et al. 27,040,690
cg18092474	15	75,019,302	*CYP1A1*	0.041 (0.010)	1.2 × 10^− 4^	0.003	0.051 (0.003)	Joubert et al. 27,040,690
cg12803068	7	45,002,919	*MYO1G*	0.060 (0.016)	2.8 × 10^− 4^	0.007	0.070 (0.003)	Joubert et al. 27,040,690
cg12101586	15	75,019,203	*CYP1A1*	0.034 (0.009)	4.1 × 10^− 4^	0.010	0.045 (0.003)	Joubert et al. 27,040,690

*Chr* chromosome; Estimate: regression coefficient from association analysis; se: standard error; Adjusted P: Bonferroni-corrected p value. Analyses were adjusted for maternal age, sex, gestational age, study centre and cellular composition. The estimates are expressed as the average difference in methylation beta values between smokers and non-smokers mothers

While no site survived Bonferroni correction for multiple testing in the single study analyses of birthweight, 3 sites showed significant negative association in meta-analysis after Bonferroni correction. No significant heterogeneity was detected between CHILD and START in this analysis, therefore fixed-effect estimates are reported (Table 4)

**Table 4:** Replicated birthweight-associated sites in meta-analysis

Site	Chr	Position	Gene	Estimate (CI)	Nominal P value	Adjusted P value	Literature estimate (CI)	Author (PMID)
cg26995690	13	36,346,376	*DCLK1*	-13.8 (-22.4, -5.2)	0.0017	0.020	-48.7 (-62.9, -34.5)	Kupers et al. 31,015,461
cg00637745	2	121,497,334	-	-35.9 (-58.6, -13.3)	0.0019	0.023	-20.9 (-26.8, -15.1)	Kupers et al. 31,015,461
cg07133097	2	121,497,538	-	-21.9 (-35.8, -7.9)	0.0021	0.025	-29.2 (-37.5, -20.8)	Kupers et al. 31,015,461

Chr: chromosome; CI: confidence interval; Adjusted P value: Bonferroni-corrected p value; Published estimate: coefficients from Kupers at al birthweight meta-analysis of all ancestries (PMID: 31,015,461). Analyses were adjusted for maternal age, sex, maternal smoking, gestational age, study centre and cellular composition. The estimates are expressed as the change in methylation beta values per one unit change in birthweight (grams)

There were no significant associations between START methylation and maternal plasma folate levels at the 48 sites from the Joubert et al. study [23]. Similarly, none of the identified GDM probes exhibited significant association with GDM in our data. All replication results including null associations are provided in Additional Table 6

## Discussion

In this report, we show that the cord blood EWAS literature based on individual CpG probe methylation differential in relation with our prenatal exposures of interest, is limited in the number of publications, as well as in the replication of associated probes. Out of the 101 publications retrieved, nineteen articles reported cord blood EWAS with significant differentially methylated sites. Most the published significant probes were only found in one study. Only 19 out of 290 probes replicated in at least another study, and all of the replicated probes were associated with maternal smoking.

CHILD and START EWAS yielded no association which survived statistical correction, except for probes associated with gestational age. However, meta-analysis of the 2 CHILD and START identified 3 significant birthweight sites.

While the use of methylation arrays allows for hypothesis-free investigations, it requires greater number of samples in order to have enough statistical power to detect effects after correcting for the multiple hypotheses testing. Many published studies have shown interesting associations between metabolic maternal measures and cord blood methylome but were underpowered [24]. Our literature review, which aimed to identify robust differentially methylated sites associated with exposures of interest and perinatal traits, showed that among hundreds of associated probes, only 19 replicated in more than one study, all of which are associated with maternal smoking. In the subset of exposure/traits we explored in our study, we showed that 4 exposures/traits show strong associations with DNA methylation: maternal smoking, maternal plasma folate levels, gestational age and birthweight, as defined by statistical significance in EWAS with greater than 50 cases and using Bonferroni correction for multiple testing. While the folate and birthweight sites originated from meta-analyses of multiple studies ^13,14^, the 86 GDM-associated probes we identified were from a small EWAS study of 88 cases [25], where FDR was used for correction (instead of Bonferroni). This study reported some FDR-corrected findings of small effect sizes.

Guided by the literature identified methylation sites, we were able to replicate associations at sites related to maternal smoking, gestational age, and birthweight, but not for GDM or plasma folate levels. Cigarette smoking was one of the strongest influential factors for the methylome as compared to other exposures. Its modulation of DNA methylation and gene expression seems to persist over time and is associated with a variety of negative health outcomes [26].

The plasma folate study from which we extracted probes, was performed in European subjects, whereas the START subjects are of South Asian descent. This may explain the lack of replication, as DNA methylation of certain areas of the genome does vary across different ancestral populations, due to a combination of different allele frequencies and complex gene-environment interactions [27–29]. Ethnicity has also been documented to be an independent determinant of folate status in a study which controlled for folate intake [30]. Canadian South-Asian women in particular have been found to have high levels of B12 deficiency compared to women of other ethnic backgrounds; and B12 and folate are metabolically inter-dependent [31].

A recent systematic review of preterm birth cohorts included only ethnically homogeneous populations (i.e. Europeans), preventing comparisons between ethnically diverse epigenomes [32]. In a replication study of the association of chronic diseases with published differentially methylated sites in 120 Batswana South African men, 86% of the associations replicated in terms of consistent size and direction of effect (but not significant p values). There were 14% of African population-specific associations which were not previously observed in European published sites [33]. Ethnic-specific methylation changes were also observed in smoking sites [34] and in cancer pathway regions at birth [35]. The epigenetic clock (epigenetic biomarkers of aging) has been proposed as a way to elucidate disease burden differential in different population since it is strongly associated with genetic race/ethnicity [36]. However, this association differs when using different epigenetic clocks, comprised of different CpG sites [37]; this warrants continued research efforts in elucidating the epigenetic/genetic race association. Using 3 different epigenetic clocks, it was shown that African-Americans generally have more wide-spread methylation changes than white Caucasians and that sex also interacts with genetic race in determining epigenetic age [38]. Together, these data certainly warrant more studies of differential methylation profiles in non-white subjects and the relationship of methylation profiles with environmental exposures in different populations.

The DOHaD framework stipulates the importance of pre-conception and early life in future health outcomes. Maternal obesity and diabetes, for instance, are known risk factors for poor fetal outcomes such as fetal/infant death, low birthweight, impaired lipid profiles and higher BMI in childhood [39–41]. Animal and human studies (including extreme food deprivation studies) have shown that DNA methylation is a major mechanism by which intrauterine and environmental exposures influence fetal development and future child health [6, 42, 43].

The life course of non-communicable disease highlights an opportune period in early life where interventions could have a high impact on life-long health and disease [5]. This early ‘plasticity’ period is the target study period of birth cohorts. A challenge is the need for well-phenotyped birth cohort with ample sample size. We previously summarized our findings on the effect of maternal and infant nutrition on cardiometabolic risk, and developed a harmonization tool to enable standardized nutrition research in different birth cohorts [44].

Strengths and Limitations: Strengths of our study include: (a) our interrogation of probes was guided by the literature to reduce number of statistical tests, (b) testing for replication in two cohorts including one of South Asian ethnicity, and (c) specificity of the START GDM phenotype which was ascertained directly by glucose oral tests, which increases the accuracy of the exposure. A main limitation of our study is the statistical power. The number of subjects of START and CHILD included in the EWAS was relatively small, which likely contributes to the few sites identified in our analysis. Another limitation of our study is the use of beta values instead of M-values for analysis of differential methylation using regression models. Despite our use of the SWAN normalization to correct noise in beta values, our results may be influenced by heteroscedasticity of beta-values of probes in the high and low methylation range. In addition, enrichment analysis of the gestational age results was performed using the Enrichr method which may be biased due to uneven representation of probes in the DNA methylation array, hence the lack of significant enrichment results may be due to this bias. Lastly, we used a reference-free unsupervised method for adjusting differential methylation analysis for cell composition, ReFACTor which was shown to improve performance on the Houseman cord blood reference dataset [20]. A good reference-based alternative would be to combine 4 MACS and FACS-sorted datasets and use the Identifying Optimal Libraries (IDOL) filter as described here [45]; which could have improved performance of the cell composition adjustment.

## Conclusions

Our study confirms the strong effect of maternal smoking during pregnancy on the infant’s methylome. Gestational age is also highly associated with differential methylation in 2 independent cohorts of different ethnicity, as well in their meta-analysis. Some previously identified birthweight sites were replicated in the meta-analysis of our 2 cohorts. There is a clear need for larger studies in diverse populations in order to establish the real effects of *in utero* exposures on infant and child health. Published EWAS associations should be reviewed carefully, for sample size and statistical correction; as well as compared to other studies to increase confidence in existing data.

## Supplementary Information

**Additional file 1.** Supplementary information. This file contains a description of the NutriGen alliance studies,data and samples collection details and sample and probe quality control procedures.

**Additional file 2.** List of associated probes from the literature search. Information about the total methylation sites in the published literature.

**Additional file 3.** List of studies identified in literature search. Details of all studies identified in literature search.

**Additional file 4.** Gene set enrichment analysis for gestational age associated genes. Results of gene set enrichment analysis for genes associated with gestational age in meta-analysis of CHILD and START.

**Additional file 5.** Results of EWAS meta-analysis of gestational age. A list of significant gestational age meta-analysis association results.

**Additional file 6.** Meta-analysis association of Gestational age with methylation in CHILD and START. Results of meta-analysis association analysis of Gestational age in CHILD and START with methylation in sites identified in the literature.

**Additional file 7.** Replication association results. Significant and null results from replication efforts for all traits.

**Additional file 8.** Volcano plot of gestational age EWAS results. Volcano plot showing gestational age EWAS meta-analysis results. Red horizontal line shows the Bonferroni correction threshold. The Y axis represents -log10 of the P values. The Y axis represents the regression coefficient of the association analysis of methylation change. *No correction for cell composition; no significant findings; gestational clock panels; sex-stratified analysis; no individual CpG analysis.

## Data Availability

All relevant data are within the paper and its Additional Information files. Raw data cannot be shared publicly because of lack of participant consent but are available upon reasonable request from the corresponding author.

## Abbreviations

BMI: body mass index
CHILD: Canadian healthy infant longitudinal development
Dohad: Developmental Origins of Health and Disease
EWAS: epigenome-wide association study
FDR: false discovery rate
FFQ: food frequency questionnaire
GDM: gestational diabetes mellitus
START: South Asian birth cohort
T2DM: type-2 diabetes mellitus
