# Supplementary material for: DNA methylation changes in cord blood and the developmental origins of health and disease – a systematic review and replication study
Source: BMC Genomics. 2022 Mar 19;23:221. doi: 10.1186/s12864-022-08451-6 (PMC8933946; doi:10.1186/s12864-022-08451-6)
Supplement: Supplementary file 1 — Additional file 1. Supplementary information. This file contains a description of the NutriGen alliance studies,data and samples collection details and sample and probe quality control procedures. [file 12864_2022_8451_MOESM1_ESM.doc]

## The NutriGen alliance

The Nutrigen alliance is comprised of 4 Canadian birth cohorts: The Family Atherosclerosis Monitoring In Early life (FAMILY), the Canadian Healthy Infant Longitudinal Development (CHILD) study, the South Asian birth Cohort (START) and the Aboriginal Birth Cohort (ABC).

1. The FAMILY cohort is designed to study determinants of CVD and is comprised of 859 mothers/901 infants, primarily white Caucasian, from Southwestern Ontario. They were recruited between 2004 and 2009 with long-term (5+ year) follow-up.
2. The CHILD study is a longitudinal birth cohort study based out of four Canadian centres. 3,600 mother/child pairs were recruited between 2008 and 2012 with the primary goal of investigating environmental and genetic determinants of allergic disorders.
3. START began recruiting in 2011 in urban Canada as well as rural and urban Bangalore in order to study environmental, genetic, and epigenetic influences on adiposity, growth, and cardio-metabolic factors in a birth cohort of South Asian women.
4. The ABC Cohort enrolled pregnant mothers from the Six Nations Reserve in Ontario. Beginning in 2012, women were recruited to study the determinants of cardiometabolic health and T2DM in an Aboriginal population.

All cohorts administered a food frequency questionnaire (FFQ) to assess prenatal diet and collected anthropometric and other health measures of the mother and infant throughout pregnancy and early childhood. These variables have been harmonized across cohorts for comparisons.

Cord blood was also collected at birth for genetic and cardiometabolic analysis. A total of 512 START and 511 CHILD were selected from their respective cohorts and randomized across arrays for methylation assessment.

For EWAS analysis, we used the following variables:

Maternal nutrition variables

The FFQ data in START was collected on a previously developed and validated South Asian Food Frequency Questionnaire [http://southasianbirthcohort.com/cms/Media/file/START_Mother_Baseline_FFQ_25April2012.pdf], and for CHILD using the Fred Hutchinson FFQ. [Fred Hutchinson Cancer Research Center. Food Frequency Questionnaires (FFQ). sharedresources.fredhutch.org. http:// sharedresources.fredhutch.org/services/food-frequencyquestionnaires-ffq]

Principal component (PC) analysis of FFQ data was used to derive individual scores representing dietary patterns. Vegetarianism was represented as a dietary pattern in these models as well as a binary (yes/no) variable. Individual nutrient information regarding fatty acid intake was used, including polyunsaturated fatty acids (PUFAs), saturated fats, and the ratio of PUFAs to saturated fats (P:S).

Two diet quality scores, the modified Alternative Healthy Eating Index (mAHEI) and the DOHaD score developed by the NutriGen group, were calculated and used in analysis.

Dietary variables were adjusted for total energy consumption when applicable.

Maternal glycemic control variables

Both START and CHILD cohorts had gestational diabetes mellitus (GDM) data available.

Additionally, in START, oral glucose tolerance test (OGTT) and AUC glucose test results were available as measures of maternal glycemic control. It was modeled as both a continuous variable and a binary variable based on a cut-off of 835. The OGTT results also allowed us to conclude participants’ GDM status using the previously described cut-offs developed in the Born in Bradford cohort for South Asian women (Farrar et al., 2015). Maternal smoking and pre-pregnancy BMI were available in CHILD only (START had no smokers).

## Sample processing and methylation probes quality control

Details on sample collection and study design for CHILD and START are outlined in the following publications:

- Moraes et al. The Canadian Healthy Infant Longitudinal Development Birth Cohort Study: Biological Samples and Biobanking. Paediatr Perinat Epidemiol. 2015; 29 (1): 84–92

- Anand SS et al. Rationale and Design of South Asian Birth Cohort (START): a Canada-India Collaborative Study. BMC Public Health. 2013;13 (1): 79

Raw methylation data from iScan were imported into R version 3.2.0 with the minfi package, which was used for all quality control and pre-processing. Samples from the START and CHILD cohorts were processed separately. Sample quality was assessed in each cohort first based on missingness criteria; any samples with a proportion of failed probes > 0.01 was removed from analysis (a total of 2 samples in START and 14 in CHILD). The getSex function in minfi was also used to estimate biological sex in each sample based on the methylation patterns of the X and Y chromosomes. This was compared to the reported sex and inconsistent samples were removed. In total 5 sample were removed from START and 7 from CHILD based on these criteria. A final sample of 506 individuals in START and 511 in CHILD remained.

Probe quality was determined with missingness criteria based on probe failure in > 0.05 of samples. Any probes exceeding this threshold were excluded: 756 from START, 634 from CHILD. In addition, all probes known to contain a SNP (70 889) or demonstrate cross-reactivity (29 233) were also removed from analysis. We assessed and corrected for batch effects (by methylation chip) using the ComBat algorithm in the sva package (Leek et al. Bioinformatics 2012). The subset within-array normalization (SWAN) method was used to normalize the methylation data to reduce differences in beta value distribution between probe types and adjust for dye bias.

A final dataset of 393 400 probes in START and 393 449 probes in CHILD remained following these quality control measures.
