# Supplementary figures and images for: DNA methylation changes in cord blood and the developmental origins of health and disease – a systematic review and replication study
Source: BMC Genomics. 2022 Mar 19;23:221. doi: 10.1186/s12864-022-08451-6 (PMC8933946; doi:10.1186/s12864-022-08451-6)

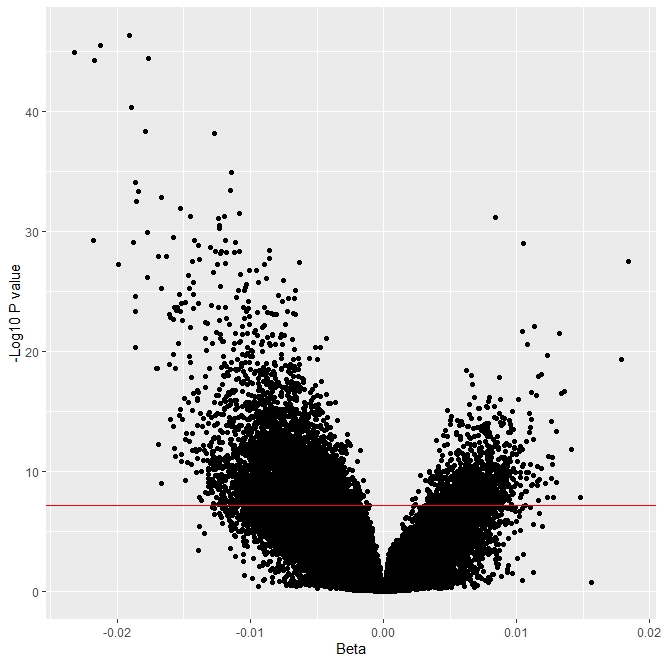

Supplement: Supplementary file 8 — Additional file 8. Volcano plot of gestational age EWAS results. Volcano plot showing gestational age EWAS meta-analysis results. Red horizontal line shows the Bonferroni correction threshold. The Y axis represents -log10 of the P values. The Y axis represents the regression coefficient of the association analysis of methylation change. *No correction for cell composition; no significant findings; gestational clock panels; sex-stratified analysis; no individual CpG analysis. [file 12864_2022_8451_MOESM8_ESM.jpeg]
